# Endothelial Ribonuclease 1 in Cardiovascular and Systemic Inflammation

**DOI:** 10.3389/fcell.2020.576491

**Published:** 2020-09-04

**Authors:** Katrin Bedenbender, Bernd T. Schmeck

**Affiliations:** ^1^Institute for Lung Research, Universities of Giessen and Marburg Lung Center, Marburg, Germany; ^2^Department of Pulmonary and Critical Care Medicine, Department of Medicine, University Medical Center Giessen and Marburg, Philipps-University Marburg, Marburg, Germany; ^3^Member of the German Center for Lung Research, Member of the German Center for Infectious Disease Research, Marburg, Germany; ^4^Center for Synthetic Microbiology, Philipps-University Marburg, Marburg, Germany

**Keywords:** ribonuclease 1, endothelial cells, inflammation, extracellular RNA, vascular diseases

## Abstract

The vascular endothelial cell layer forms the inner lining of all blood vessels to maintain proper functioning of the vascular system. However, dysfunction of the endothelium depicts a major issue in context of vascular pathologies, such as atherosclerosis or thrombosis that cause several million deaths per year worldwide. In recent years, the endothelial extracellular endonuclease Ribonuclease 1 (RNase1) was described as a key player in regulation of vascular homeostasis by protecting endothelial cells from detrimental effects of the damage-associated molecular pattern extracellular RNA upon acute inflammation. Despite this protective function, massive dysregulation of RNase1 was observed during prolonged endothelial cell inflammation resulting in progression of several vascular diseases. For the first time, this review article outlines the current knowledge on endothelial RNase1 and its role in function and dysfunction of the endothelium, thereby focusing on the intensive research from recent years: Uncovering the underlying mechanisms of RNase1 function and regulation in response to acute as well as long-term inflammation, the role of RNase1 in context of vascular, inflammatory and infectious diseases and the potential to develop novel therapeutic options to treat these pathologies against the background of RNase1 function in endothelial cells.

## Introduction

Endothelial cells (ECs) form the inner lining of all blood vessels and act as anatomical and active physiological barrier to separate the blood from the surrounding tissue (Baldwin and Thurston, 2001; Michiels, 2003). Thereby, these cells highly contribute to control and maintenance of vascular homeostasis and integrity (Pober and Sessa, 2007; Rajendran et al., 2013). Under physiological conditions, the endothelium is the central component of vessel permeability and participates in the regulation of coagulation as well as the communication with band recruitment of circulating leukocytes from the blood stream (Arnout et al., 2006; Ley and Reutershan, 2006; Minshall and Malik, 2006; Pober and Sessa, 2007). In this regard, ECs sequester leukocyte interactive proteins, such as chemokines or adhesion molecules within their storage vesicles, the Weibel-Palade Bodies (WPBs), and additionally repress the transcription of membrane bound adhesion molecules or proinflammatory cytokines (De Caterina et al., 1995; Rondaij et al., 2006; Pober and Sessa, 2007; Rajendran et al., 2013). Upon inflammation, ECs get rapidly activated, which goes along with decisive changes in gene expression, e.g., cytokines and adhesion molecules, WPB exocytosis to promote the release proinflammatory agents into the extracellular space, and exposure of adhesion molecules at the cell surface. Consequently, these processes support recruitment and interaction with circulating leukocytes, finally resulting in infiltration of inflammatory cells into the underlying tissue and secretion of proinflammatory mediators such as tumor necrosis factor alpha (TNF-α) or interleukin (IL)-1β into the blood stream. Altogether, these processes act in concert to eradicate the triggering inflammatory stimulus and restore vascular and tissue integrity (Pober and Sessa, 2007). However, persistent vascular inflammation can drastically affect the homeostatic function of the endothelium, followed by EC dysfunction and progression of vascular diseases, such as atherosclerosis, thrombosis, or consequential disorders like myocardial infarction or cerebral ischemia (Poredos, 2002; Sitia et al., 2010; Zernecke and Preissner, 2016). Such vascular diseases depict one of the leading causes of death worldwide with approximately 18 million deaths per year^1^. In recent decades, investigation of the underlying mechanisms of EC dysfunction in inflammation-associated vascular diseases and the development of novel therapeutic approaches to treat these pathologies was of great importance. In this context, Ribonuclease (RNase) 1 has been newly recognized as a vessel-protective factor in EC inflammation that is tightly associated to inflammation induced vascular dysfunction and subsequent pathologies (Zernecke and Preissner, 2016).

This review article summarizes the current knowledge of endothelial RNase1 and its regulation and function in vascular ECs under physiological and inflammatory conditions. Moreover, we outline the impact of RNase1 regulation in progression of diverse vascular diseases, as well as other inflammation-associated disorders, and the resulting potential to develop novel therapeutic strategies to treat pathological EC inflammation by preserving RNase1 function.

## Endothelial RNase1

RNase1 belongs to the Ribonuclease A superfamily, consisting of in total 13 described enzymes (Sorrentino, 2010; Koczera et al., 2016). The biological function of this enzyme family varies from host defense and immune cell regulation [e.g., RNase2, RNase5, RNase7; (Hooper et al., 2003; Yang et al., 2004; Bedoya et al., 2006; Huang et al., 2007)] to tissue repair and remodeling, such as angiogenesis [e.g., RNase4, RNase5, RNase7; (Fett et al., 1985; Li et al., 2013; Schwartz et al., 2018)], as well as RNA cleavage [e.g., RNase5; (Lee and Vallee, 1989; Russo et al., 1994)]. Nevertheless, the precise physiological functions of the diverse members of the Ribonuclease A superfamily need to be further investigated (Koczera et al., 2016). The RNase1 endonuclease is described as one of eight secreteable and enzymatically active members of this protein family, also known as canonical RNases (Koczera et al., 2016). Thermostable RNase1 depicts the most prevalent human homolog of the well-described bovine pancreatic RNase A and was shown to be produced in numerous tissues of the whole body, such as pancreas, testis, ovary, and brain (Beintema et al., 1988; Futami et al., 1997; Landre et al., 2002; Sorrentino, 2010; Koczera et al., 2016). Moreover, human RNase1 mRNA and protein expression data from high-throughput analysis provided by the “proteinatlas” platform^2^ is summarized in Table 1. Thereby, high levels of active RNase1 can be detected in different body fluids, e.g., plasma or serum, with concentrations up to 0.5 μg/ml, suggesting an endothelial origin of this enzyme (Weickmann et al., 1984; Morita et al., 1986; Futami et al., 1997; Petrova et al., 2002; Su et al., 2004). Indeed, RNase1 expression and release is predominantly observed in various types of vascular ECs for instance in primary human ECs from pulmonary arteries, cerebral microvasculature, or especially human umbilical vein ECs (HUVEC), that secrete highest amounts of RNase1 (Landre et al., 2002; Fischer et al., 2011). Thereby, these cells offer an ideal model system to study RNase1 function and regulation in human ECs and are widely used for further investigations.

**TABLE 1:** RNase1 mRNA and protein expression in human tissue https://www.proteinatlas.org/ENSG00000129538-RNASE1/tissue.

Tissue		mRNA	Protein
Brain	Olfactory region, amygdala, hypothalamus, thalamus, midbrain, pons and medulla, corpus callosum, spinal cord	yes	n/a
	Cerebral cortex, hippocampal formation, basal ganglia	yes	Neuronal cells
	Cerebellum	yes	Purkinje cells
Eye	Retina	yes	n/a
Endocrine tissue	Thyroid gland, parathyroid gland, adrenal gland	yes	n/d
	Pituitary gland	yes	n/a
Lung	Nasopharynx	n/a	n/d
	Bronchus	yes	Respiratory epithelial cells
	Lung	yes	n/d
Proximal digestive tract	Tongue	yes	n/a
	Oral mucosa	n/a	Squamos epithelial cells
	Salivary gland	yes	Glandular cells
	Esophagus	yes	n/d
Gastrointestinal tract	Stomach, duodenum, small intestine, colon, rectum	yes	Glandular cells
Liver and gallbladder	Liver, gallbladder	yes	n/d
Pancreas	Pancreas	yes	Exocrine glandular cells
Kidney and urinary bladder	Kidney	yes	Cells in tubules
	Urinary bladder	yes	n/d
Male tissues	Ductus deferens	yes	n/a
	Testis	yes	Cells in seminiferous duct
	Epididymis, seminal vesicle	yes	Glandular cells
	Prostate	yes	n/d
Female tissues	Vagina, ovary, fallopian tube, endometrium, cervix, uterine, placenta, breast	yes	n/d
Muscle tissues	Heart muscle, smooth muscle, skeletal muscle	yes	n/d
Adipose and soft tissue	Adipose tissue	yes	n/d
	Soft tissue	n/a	Chondrocytes
Skin	Skin	yes	n/d
Bone marrow and lymphoid tissues	Thymus	yes	n/a
	Appendix	yes	Glandular cells
	Spleen, lymph node, tonsil	yes	n/d
Blood	Bone marrow	yes	n/d
	Granulocytes, monocytes, dendritic cells, total PBMCs	yes	n/a

*n/a, not applicable, n/d, not detected, PBMCs, peripheral blood derived mononuclear cells; Data provided by www.proteinatlas.org.*

### RNase1 Glycosylation and Its Storage in WPBs

Secreted RNase1 molecules vary in size from approximately 18–26 kDa, depending on its glycosylation pattern (Landre et al., 2002; Koczera et al., 2016; Ressler and Raines, 2019). Investigation of RNase1 isolates from human serum identified exclusively three different N-glycosylation sites at secreted proteins with varying abundance and differential expression depending on tissue and cell type: Asparagine (Asn) 34, Asn76 and Asn88, with Asn34 presumably being the most important glycosylation sites resulting in significantly improved overall protein stability and robust catalytic activity (Beintema et al., 1988; Ribo et al., 1994; Barrabes et al., 2007; Ressler and Raines, 2019). Despite the function of RNase1 protein glycosylation in respect to stability and catalytic activity, further consequences of RNase1 glycosylation are still unknown (Ressler and Raines, 2019). In general, glycosylation is already intensively studied, for instance in context of protein stability, dictation of immune cell movement, or discrimination between self and non-self (Marth and Grewal, 2008; Reily et al., 2019; Ressler and Raines, 2019). Since, studies of the Ribonuclease A superfamily members RNase2 and RNase3 indicated a crucial role of their N-glycosylation in host-defense (Sorrentino, 2010; Lu et al., 2018), a comparable feature for RNase1 glycosylation in this context can be suggested. As protein glycosylation is a common co-translational modification originated from the endoplasmatic reticulum and the Golgi apparatus, it is hardly surprising that secreted RNase1 is partially stored and released from WPBs (Marth and Grewal, 2008; Fischer et al., 2011; Reily et al., 2019). WPBs are small storage granules of vascular ECs and are known to be the “first aid kit” of the endothelial cell layer. These granules consist of several proinflammatory mediators such as adhesion molecules (e.g., P-selectin) or chemo - and cytokines (e.g., IL-8, monocyte chemoattractant protein 1) as well as the potent coagulation factor von Willebrand factor, which guides manufacturing and loading of mature WPBs at the trans-Golgi network (Valentijn et al., 2011; Schillemans et al., 2019). In respect to RNase1, Fischer and colleagues demonstrated that constitutively expressed endothelial RNase1 can be stored, and released from WPBs, where it co-localizes with factors like von Willebrand factor or P-selectin (Fischer et al., 2011; Koczera et al., 2016). Although the knowledge about WPB-assembly and loading has considerably increased in the past years, the exact mechanism by which RNase1 enters these vesicles is still unclear. Nevertheless, once packed with cargo, WPBs travel to the cell periphery and can release their content, including RNase1, to the extracellular space under physiological as well as inflammatory conditions (Fischer et al., 2011; Valentijn et al., 2011; Schillemans et al., 2019).

### RNase1 Ribonuclease Function and Inhibition

According to its secretory phenotype, RNase1 comprises the ability to act in many body fluids, which is tightly associated to its neutral pH optimum to provide enhanced capacity to cleave single- as well as double-stranded RNA species in the extracellular space (Sorrentino et al., 2003; Koczera et al., 2016; Lomax et al., 2017). The catalytic mechanism is based on the interaction of cationic enzymatic residues with the anionic phosphoryl groups of RNA substrates and the formation of disulfide bonds (Klink et al., 2000; Ressler and Raines, 2019).

Thereby, RNase1 can act as an RNA scavenger to modulate and remove the heterogeneous content of extracellular RNA (eRNA) that is highly associated to vascular inflammation (Koczera et al., 2016; Zernecke and Preissner, 2016; Lu et al., 2018). With respect to the high affinity of RNase1 to diverse RNA species, it is necessary to prevent intracellular self-RNA degradation, as well as excessive degradation of damage-associated RNA species in the extracellular space to maintain cellular responsibility. Intracellularly, RNases, including RNase1, can be efficiently bound by the human RNase inhibitor to prevent cytotoxicity by blocking the catalytic activity of the RNase via formation of a tight RNase-inhibitor complex. Thereby, the 50 kDa, cytosolic and anionic RNase inhibitor effectively binds the cationic surface of the enzyme via formation of several intermolecular hydrogen bonds to provide an extremely stable complex and protect intracellular RNA species from degradation (Dickson et al., 2005; Johnson et al., 2007). In terms of RNase1 clearance from the extracellular space, RNases can be taken up and removed from the extracellular space via the endocytic pathway by neighboring cells, however, the precise mechanisms need to be further investigated (Chao and Raines, 2011; Lomax et al., 2017).

## RNase1 Regulation Upon EC Inflammation

The release of glycosylated RNase1 from WPBs of vascular ECs, its high secretory levels into body-fluids, and its catalytic activity to diverse eRNA species pointed toward a regulatory impact of RNase1 on maintenance and integrity of vascular homeostasis (Landre et al., 2002; Sorrentino et al., 2003; Kannemeier et al., 2007; Fischer et al., 2011; Lomax et al., 2017). Accordingly, in 2014, Gansler and colleagues analyzed the regulation of RNase1 in context of acute and long-term EC inflammation, thereby revealing its importance as regulator of vascular function (Gansler et al., 2014).

### The RNase1-eRNA System in ECs

In response to inflammation, infection or tissue injury, ECs get activated and initiate the release of leukocyte interactive molecules as well as large amounts of free damage-associated molecular patterns such as eRNA, e.g., via apoptosis or necrosis (Pober and Sessa, 2007; Fischer and Preissner, 2013; Fischer et al., 2013; Zernecke and Preissner, 2016). Once released to the extracellular space, free eRNA can act as potent inducer of the immune response, for instance via signaling through pattern-recognition receptors such as endosomal Toll-like receptors (reviewed by Fischer and Preissner, 2013; Fischer et al., 2014; Zernecke and Preissner, 2016; Lu et al., 2019), and the secretion of WPB-content from the EC layer (Fischer et al., 2013, 2014; Gansler et al., 2014; Zernecke and Preissner, 2016). These processes subsequently result in the release of the proinflammatory content of WPBs, including RNase1. Here, RNase1 protects the endothelium from an overwhelming eRNA-mediated inflammatory response by acting as natural counterpart to eRNA via degradation and generation of eRNA cleavage-products that are unable to disturb the EC-barrier function (Fischer et al., 2007, 2014; Cabrera-Fuentes et al., 2014; Gansler et al., 2014). However, upon prolonged EC inflammation, the balance of the RNase1-eRNA system is compromised due to accumulation of eRNA in the extracellular space. Thereby, eRNA facilitates various processes affecting the integrity of the endothelium: eRNA exposure of several types of vascular ECs increased vessel permeability via disorganization of cell-junctional proteins, like occludin or vascular endothelial cadherin. These processes are mediated via enhanced activation of vascular endothelial growth factor and vascular endothelial growth factor receptor two signaling (Fischer et al., 2007, 2009; Gansler et al., 2014). Additionally, augmented secretion of proinflammatory cytokines by circulating immune cells (e.g., monocytes, macrophages) is induced via eRNA-mediated signaling through pattern recognition receptors or cytokine shedding (Fischer and Preissner, 2013; Fischer et al., 2013, 2014; Gansler et al., 2014; Zernecke and Preissner, 2016; Lu et al., 2019), phospholipase C function, and intracellular Ca^2+^ release (Fischer et al., 2009). All these processes culminate in a highly proinflammatory state of the endothelium that is associated to a vast increase in cytokine levels in the extracellular space, especially TNF-α or IL-β. These cytokines further act on ECs via massive repression of RNase1 expression, as demonstrated by Gansler and colleagues in HUVEC (Gansler et al., 2014). Consequently, RNase1 protein generation, storage, and release from WPBs, as well as its eRNA-degrading function is impaired, followed by additional eRNA accumulation in the extracellular space and subsequent EC dysfunction (Gansler et al., 2014).

### Molecular Mechanisms of Proinflammatory RNase1 Repression

**FIGURE 1:**
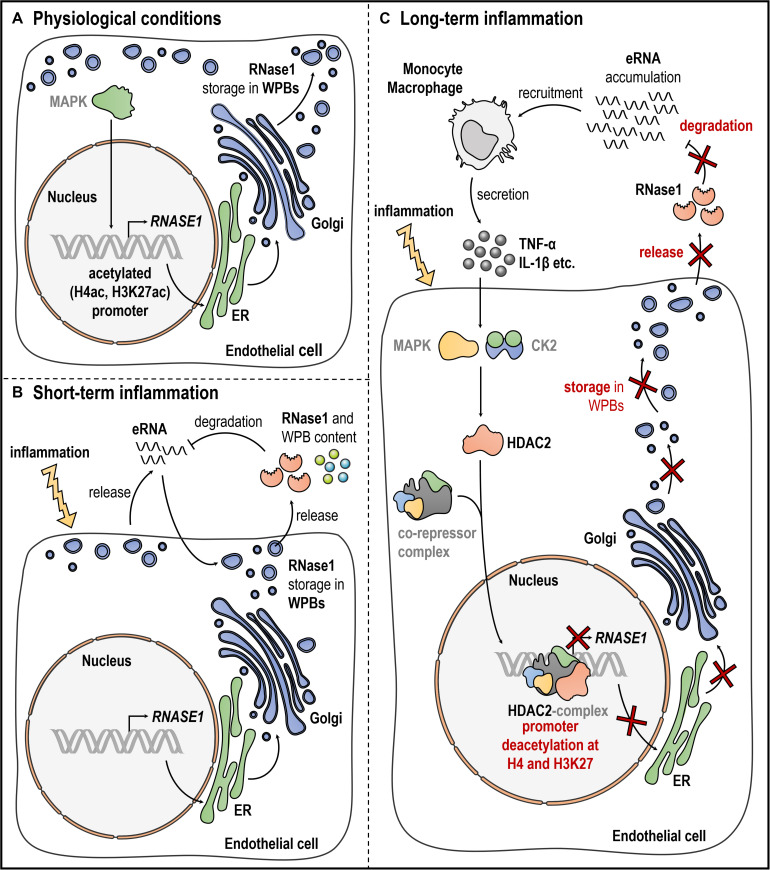
Endothelial RNase1 regulation. Schematic overview of RNase1 regulation under **(A)** physiological conditions, **(B)**, short-term, or **(C)** long-term inflammation of ECs. **(A)** Under physiological conditions, RNase1 expression might be facilitated through MAPK signaling, *RNASE1* promoter H4ac and H3K27ac along with polymerase II transcription machinery binding. RNase1 protein and other proinflammatory/prothrombotic agents are stored in WPBs that are generated at the ER-Golgi network and located at the cell membrane. **(B)** Upon short-term inflammation, ECs get activated resulting in eRNA release to induce the immune response. eRNA initiates exocytosis of WPBs, releasing their content, including RNase1, into the extracellular space. RNase1 acts as counterpart to eRNA by degradation to protect the cells from detrimental eRNA-mediated effects. **(C)** Upon long-term inflammation, eRNA accumulates in the extracellular space, recruiting inflammatory cells (e.g., monocytes, macrophages) that secrete high amounts of proinflammatory agents like TNF-α or IL-1β. These cytokines act on RNase1 expression presumably via activation MAPK signaling and CK2-mediated signaling, mounting HDAC2 binding to so far unknown co-repressor complex(es), accumulation at the *RNASE1* promoter, H4 and H3K27 deacetylation and subsequent gene repression. CK2: casein kinase two, EC: endothelial cell, ER: endoplasmatic reticulum, eRNA: extracellular RNA, HDAC2: histone deacetylase 2, H4ac: histone four acetylation, H3K27ac: histone three lysine 27 acetylation, IL-1β: interleukin 1 beta, MAPK: mitogen-activated protein kinase, RNase1: Ribonuclease 1, TNF-α: tumor necrosis factor alpha, WPBs: Weibel-Palade Bodies. Gray writing depicts presumably involved molecules. This figure was created with the help of smart Servier Medical Art https://smart.servier.com/.

In recent years, the identification of the molecular mechanisms of inflammation-mediated RNase1 regulation was intensively studied, yielding in a substantial increase in knowledge. In 2014, Gansler and colleagues implicated a regulatory mechanism for RNase1 repression, dependent on histone deacetylases (HDACs) rather than Nuclear factor κ B-mediated signaling by using specific pharmaceutical inhibitors (Gansler et al., 2014). Based on these findings, we further investigated the precise molecular mechanisms of RNase1 regulation: The promoter region of *RNASE1* was identified to examine the HDAC-mediated changes of *RNASE1* at chromatin level (Bedenbender et al., 2019). Thereby, proinflammatory stimulation of HUVEC resulted in the loss of different markers associated to active transcription, specific deacetylation of the *RNASE1* promoter at histone 4 and histone 3 lysine 27, along with the loss of RNA polymerase II transcription machinery binding (Kouzarides, 2007; Wang et al., 2008; Bedenbender et al., 2019). Additionally, administration of the specific HDAC inhibitor MS275, targeting the class I HDACs HDAC1–3, successfully recovered RNase1 mRNA expression upon inflammation (via TNF-α) as consequence of restored histone acetylation and RNA polymerase II recruitment (Bedenbender et al., 2019). The HDAC enzyme responsible for this regulatory process was further identified as HDAC2, demonstrated by its accumulation and deacetylation of the *RNASE1* promoter upon proinflammatory stimulation. However, the results also implicated that the highly similar class I family member HDAC1 might act redundantly to HDAC2 in this context (Sengupta and Seto, 2004; Yang and Seto, 2008; Bedenbender et al., 2019). In respect to the underlying signaling cascade, only little is known from literature. It was demonstrated that Nuclear factor kappa B-mediated signaling did not directly influence RNase1 expression upon proinflammatory stimulation (Gansler et al., 2014). However, it is worth to mention that the described proinflammatory agents have the potential to induce an inflammatory loop of cytokine production via Nuclear factor kappa B signaling (Wajant and Scheurich, 2011), which in turn might increase the negative impact of proinflammatory conditions on RNase1. Nevertheless, several intermediate steps in the RNase1 regulatory signaling cascade in human ECs can be speculated: RNase1 regulation in HUVEC was identified as a specific proinflammatory reaction mediated via e.g., TNF-α, IL-1β or polyinosinic polycytidylic acid (poly I:C) signaling (Gansler et al., 2014; Bedenbender et al., 2019). These results assume a regulatory mechanism by common signaling cascade(s) that is independent from Nuclear factor kappa B. Here, TNF-α, IL-1β, or poly I:C are described as potent regulators of the inflammatory response via activation of the mitogen activated protein kinase pathways, for instance via c-Jun N-terminal kinase or p38 (Kawai and Akira, 2006; Weber et al., 2010; Brenner et al., 2015). These findings support the hypothesis of a mitogen activated protein kinase-dependent RNase1 regulation in human ECs. Despite the signaling cascade, it is still unclear how HDAC2 is recruited to the *RNASE1* promoter to conduct its deacetylase function. Based on the literature HDAC2 activity might be regulated by casein kinase two via phosphorylation (Tsai and Seto, 2002; Litchfield, 2003; Brandl et al., 2009; Segre and Chiocca, 2011). This further supports HDAC2 association into multiprotein co-repressor complexes (e.g., REST co-repressor complex, SIN3 complex and Nucleosome Remodeling and Deacetylase complex) and recruitment to the *RNASE1* promoter (Zhang et al., 1997; Tong et al., 1998; You et al., 2001; Sengupta and Seto, 2004; Sun et al., 2007; Yang and Seto, 2008). An overview of the current knowledge of RNase1 regulation, also including potential intermediate steps, in EC inflammation is depicted in Figure 1.

### Other RNase1 Regulating Factors

Although the current literature mainly focuses on proinflammatory agents like TNF-α or IL-1β, it is worth mentioned that also other stimuli were investigated in respect to RNase1 regulation. The aforementioned proinflammatory stimuli as well as the double-stranded RNA analog poly I:C or prothrombotic factors like eRNA, thrombin or vasopressin, initiated RNase1 release from WPBs upon short term stimulation (Fischer et al., 2011; Gansler et al., 2014). However, upon long-term treatment, thrombin as well as a ligand for the endosomal Toll-like receptor-3, poly I:C, significantly mediated RNase1 repression in HUVEC, like TNF-α or IL-1β (Akira and Takeda, 2004; Gansler et al., 2014; Bedenbender et al., 2019). Compared to that, other proinflammatory agents like the Toll-like receptor-4 ligand lipopolysaccharide or IL-13 failed to do so, indicating a specific proinflammatory regulation of endothelial RNase1 upon certain treatments (Akira and Takeda, 2004; Bedenbender et al., 2019). Additionally, there is also indication that treatment of HUVEC with interferon gamma comprises the ability to promote RNase1 expression (Bedenbender et al., 2019). Based on these findings, it will be of future interest to not only investigate RNase1 repressive mechanisms but also RNase1 promoting factors that might offer potential new strategies to promote RNase1 restoration during resolution phase of inflammation and protect vascular integrity upon inflammation.

## Protective RNase1 Function in Diseases

### RNase1 Function in Cardiovascular Pathologies

In recent years, researchers provided strong evidence that imbalance of the RNase1-eRNA system and accumulation of nucleic acids species like eRNA contribute to development and progression of several vascular pathologies such as atherosclerosis or thrombosis. Thereby, eRNA acts not only as inducer of defense reactions by functioning as novel danger signal, but also acts as potent cofactor in context of vascular inflammation, finally counteracting RNase1 function and therewith misbalancing the RNase1-eRNA system (Zernecke and Preissner, 2016). In this context, previous studies demonstrated a protective role of RNase1 in diverse vascular as well as inflammatory disorders, focusing on the means of RNase1 administration as new therapeutic strategy to prevent disease progression.

#### Atherosclerosis and Thrombosis

Atherosclerosis is characterized by the formation of fibrous, fatty plaques in vascular walls, resulting in limited blood flow and tissue ischemia. These atherosclerotic lesions can further disrupt to provoke thrombus formation (Libby et al., 2019). Healthy ECs contribute to the regulation of coagulation and thrombus formation to maintain vascular homeostasis by secretion of both pro- and antithrombotic factors (Bochenek and Schafer, 2019). However, prolonged vessel injury and inflammation result in EC dysfunction, causing a pathological outcome of this process by complete vessel occlusion and development of consequential pathologies like myocardial infarction (MI) or stroke (Bochenek and Schafer, 2019; Libby et al., 2019). In this context, eRNA was identified to be enriched at sites of vascular injury, acting as a prothrombotic and proinflammatory factor (Zernecke and Preissner, 2016). Here, eRNA acts as a cofactor for contact phase proteins to induce the intrinsic blood coagulation cascade via factor XII/XI and promotes thrombus formation (Kannemeier et al., 2007; Fischer and Preissner, 2013). Therefore, several studies investigated the vessel protective function of RNase1 administration as a new potential therapeutic option to prevent plaque formation and vessel occlusion. Simsekyilmaz and colleagues described the accumulation of eRNA and proinflammatory mediators in atherosclerotic lesions or plasma of low-density lipoprotein receptor or Apolipoprotein E deficient mice that promote disease progression and reduction of RNase1 activity. Interestingly, administration of RNase1 decreased eRNA levels, inflammatory cell recruitment, proinflammatory mediators, and plaque formation in these animals (Simsekyilmaz et al., 2014). Additionally, prothrombotic eRNA was also associated to fibrin-rich thrombi in an arterial thrombosis mouse model and pretreatment of mice with RNase1 considerably reduced eRNA containing thrombi and delayed vessel occlusion (Kannemeier et al., 2007). These findings are in line with the phenotype of recently described RNase1 deficient mice that showed increased eRNA plasma levels and more rapid blood clotting, indicating a crucial role for RNase1 in regulation of blood coagulation (Garnett et al., 2019).

#### Myocardial Infarction and I/R Injury

As consequential disorders of atherosclerosis and thrombosis, MI and ischemia/reperfusion (I/R) injury are of great importance. In MI, damaged cardiac tissue was shown to release proinflammatory and prothrombotic mediators, like TNF-α or eRNA, contributing to I/R injury (Cabrera-Fuentes et al., 2014; Chen et al., 2014). Several *in vivo* models implicated an important function of RNase1 administration in prevention and severity reduction of MI and I/R injury. Increased release of eRNA from injured cardiomyocytes was associated to a robust cytokine response in coronary artery occlusion mouse models followed by I/R. Here, eRNA levels, myocardial cytokines, leukocyte infiltration and myocyte apoptosis were diminished upon RNase1 administration, resulting in cardiac protection (Chen et al., 2014). Moreover, similar results were obtained in mice or isolated I/R Langendorff-perfused rat heart models. eRNA and TNF-α-induced cardiomyocyte death was prevented by RNase1 administration, resulting in reduced MI size and recovery of cardiac tissue (Cabrera-Fuentes et al., 2014). Additionally, RNase1 application also reduced eRNA-mediated myocardial edema formation, infarction size, improved artery perfusion, and conclusively increased survival rates of tested animals (Stieger et al., 2017). Finally, these findings have been utilized to study the RNase1-eRNA system in context of cardiac remote ischemic preconditioning in humans to prevent acute I/R injury during cardiac surgery. These findings revealed that remote ischemic preconditioning prior to surgery increased endogenous plasma RNase1 levels to reduce circulating eRNA and TNF-α and improve surgery outcome (Cabrera-Fuentes et al., 2015).

#### Hepatic Ischemia

Besides the heart, also other organs can be affected by thrombosis and I/R injury, e.g., the liver (Ma et al., 2017). In a mouse model of hepatic ischemia reperfusion, Ma and colleagues revealed increased serum and hippocampus proinflammatory cytokines (e.g., IL-1β, IL-6) and eRNA levels, along with cognitive impairment of treated animals in a liver ischemia reperfusion model. Interestingly, also these processes were markedly decreased by RNase1 administration (Ma et al., 2017).

#### Cerebral Edema and Stroke

Additionally, thrombus formation often results in progression of cerebral edema and stroke. In this context, different RNA species e.g., eRNA, poly I:C or single-stranded RNA, have been found to be associated to impaired blood-brain-barrier function (Fischer et al., 2007; Walberer et al., 2009). *In vivo* rodent animal models of sinus saggitalis thrombosis or middle cerebral artery occlusion to induce stroke and edema formation revealed that RNase1 administration considerably diminished vessel occlusion, infarct size, and edema formation (Fischer et al., 2007; Walberer et al., 2009).

### RNase1 Function in Infectious and Inflammatory Disorders

Although studies addressing RNase1-eRNA related pathologies mainly focused on vascular diseases, there is also indication for an important function of RNase1 in several other disorders. For instance, RNase1 participated in recruitment of immune cells, in particular dendritic cells (Yang et al., 2004). These findings also suggested an important role for RNase1 in context of inflammatory or infectious diseases, thereby expanding the vessel-protective function of RNase1 beyond classical cardiovascular disorders.

#### Infectious Diseases

Human RNases are tightly associated to infectious diseases as already described for several members of the RNase A superfamily, e.g., the antiviral RNase2 (Koczera et al., 2016; Lu et al., 2018). Although less is known in this field about RNase1, there are a few publications addressing the function of RNase1 on different pathogens. In this context Bedoya as well as Rugeles and colleagues revealed an HIV inhibitory effect of RNase1 in infected lymphocytes (Rugeles et al., 2003; Bedoya et al., 2006). Additionally, the induction of RNase1 by interferon gamma (Bedenbender et al., 2019) also points toward a potential antiviral function of this enzyme that might be comparable to another RNase A superfamily member, RNaseL (also known as RNase4), that acts as an essential component of the interferon-mediated antiviral immune response (Li et al., 1998; Liang et al., 2006). Moreover, RNase1 was demonstrated to be involved in fighting *Streptococcus pneumoniae* infection of alveolar epithelial cells. Here, eRNA increased *S. pneumoniae* invasion of alveolar epithelial cells through its bacterial cell wall binding properties, while RNase1 treatment prevented this eRNA-pneumococcal interaction and subsequent infection (Zakrzewicz et al., 2016). Finally, RNase1expression was suppressed in context of other bacterial infections like *Francisella tularensis* or *Mycobacterium tuberculosis*, indicating RNase1 administration as potential therapeutic option to fight such pathogens (Lu et al., 2018). Thus, it can be speculated that RNase1 administration might be beneficial in diverse infectious contexts and should be in focus of future investigations.

#### Sepsis

Sepsis can induce dangerous organ dysfunctions and tissue injury that is associated with high levels of damage-associated molecular patterns, such as diverse types of eRNA that signal via pattern-recognition receptors, as well as severe dysregulation of cytokine production (“cytokine storm”), including TNF-α (Bianchi, 2007; Chan et al., 2012; Zechendorf et al., 2020). In context of septic cardiomyopathy, RNase1-counteracting molecules like eRNA were demonstrated to be significantly increased during sepsis and associated to cardiomyocyte apoptosis. Recent findings demonstrated that administration of RNase1 to septic mice considerably attenuated cardiac apoptosis, cytokine secretion and finally cardiomyopathy (Zechendorf et al., 2020).

#### Rheumatoid Arthritis

Chronic inflammatory rheumatoid arthritis (RA) is associated with chronic inflammation, synovial hyperplasia, and local hypoxia, resulting in synovial cell activation and massive tissue damage (Neumann et al., 2018). Within this context, activated RA synovial fibroblasts secreted high amounts of proinflammatory agents as well as eRNA, which was associated to RA fibroblast invasion into cartilage and tissue destruction (Zimmermann-Geller et al., 2016; Neumann et al., 2018). In an *in vivo* RA mouse model system, RNase1 application successfully prevented synovial fibroblast invasion, indicating that the RNase1-eRNA system contributes to RA pathophysiology (Zimmermann-Geller et al., 2016).

#### Cancer

The function of Ribonucleases as potential cancer therapy is already widely studied, due to their RNA-degrading ability and the associated potential to block protein biosynthesis, as well as the release of circulating eRNA species by tumor cells (Kopreski et al., 2001; Ardelt et al., 2009). Since, RNases are subsequently bound by RNase inhibitor upon entering the cytoplasm to prevent cytotoxicity, ongoing research focuses on development of artificial RNase variants to target cancer cells as chemotherapeutic options by increasing their cytotoxic potential (Lomax et al., 2017). Thereby, artificial RNase1 variants were designed that evade cytosolic RNase inhibitor binding to evoke cytotoxic effects (Psarras et al., 1998; Yagi et al., 2006). For instance, genetic insertion of human basic fibroblast growth factor into the RNase inhibitor binding site of RNase1 resulted in *in vivo* growth inhibition of squamous cell carcinoma in mice (Yagi et al., 2006). Additionally, previous research by Fischer and colleagues indicated a substantial role of eRNA in tumor cell migration through human cerebral microvascular ECs, including TNF-α secretion from tumor-infiltrating immune cells. Here, tumor volume and weight can be diminished by RNase1 treatment in a human subcutaneous xenograft cancer model (Fischer et al., 2013). These findings further demonstrate the potential of RNase1 as anti-cancer treatment. Besides the described ability to act as an anti-cancer drug, RNase1 is also suggested to operate as a tumor marker for pancreatic cancer, since tumor cells and cancer-associated ECs secreted a differently glycosylated form of RNase1 compared to healthy cells (Peracaula et al., 2003; Barrabes et al., 2007).

## Targeting RNase1 Repression as Novel Therapeutic Strategy in Diseases

Endothelial RNase1 was demonstrated as promising target for the development of novel treatment strategies in context of diverse pathologies, ranging from cardiovascular diseases over inflammatory and infectious pathologies to cancer. The current literature already describes several approaches that clearly define the protective function of RNase1 administration in different *in vitro* and *in vivo* models, also including at least one clinical approach in humans. However, these studies mainly focus on recovering RNase1 levels by enzyme addition instead of targeting the responsible repressive signaling cascades and molecules. Recent work revealed many aspects of the RNase1 repressive signaling pathway that is initiated by eRNA and high proinflammatory cytokine levels, like TNF-α or IL-1β. Unraveling these signaling pathways provides several potential access points for therapy. These targets might include mitogen-activated protein kinases like p38, casein kinase two, as well as HDAC2 and responsible co-repressor complex(es), which directly bind the *RNASE1* promoter to repress gene expression by chromatin remodeling. In this regard, several studies already focused on the usage of distinct pharmaceutical substances to block the function of these molecules in diverse pathologies. For instance, mitogen-activated protein kinase p38 as well as casein kinase two inhibitors have been applied in studies analyzing atherosclerosis, MI and I/R injury, or stroke (Barone et al., 2001; Zhao et al., 2002; Kaiser et al., 2004, 2005; Harvey et al., 2007), and both are used in clinical trials for diverse cancers and inflammatory disorders, e.g., RA (Kumar et al., 2003; Lee and Dominguez, 2005; Buontempo et al., 2018). Additionally, universal as well as specific HDAC inhibitors are already under clinical investigation or used for cancer therapy, by directly targeting the chromatin remodeling function of these enzymes (Yoon and Eom, 2016). Moreover, HDAC inhibitors were also described to be beneficial in context of cardiovascular diseases, like atherosclerosis or MI (Mann et al., 2007; Granger et al., 2008; Findeisen et al., 2011; Yoon and Eom, 2016). Based on these findings, further investigation of alternative strategies to prevent RNase1 repression is of great importance. Additionally, future investigations to develop novel therapeutic approaches should also include the analysis of physiological and RNase1 promoting signaling cascades and factors, such as interferon γ, to further expand and improve novel therapeutic options to preserve the vessel protective function of RNase1 in context of vascular inflammation.

## Closing Remarks and Future Perspectives

Endothelial RNase1 acts as potent vessel protective factor in context of vascular inflammation by counteracting damage-associated eRNA that is released upon vascular injury. However, long-term inflammation represses RNase1 expression and function by a mechanism where eRNA and high pro-inflammatory cytokine levels induce a molecular signaling cascade presumably via mitogen-activated protein kinase(s), casein kinase two and HDAC2-containing co-repressor complex(es) that finally ends up in histone deacetylation and transcriptional repression of RNase1. Although a large part of the RNase1 repressive signaling cascade as well as RNase1 structure and function have already been described extensively, several aspects remain unknown. Thus, it is of great importance to further define the molecular mechanism of RNase1 regulation (e.g., involved signaling pathways, HDAC2 activation, co-repressor complex(es), transcription factors, or RNase1 promoting factors), the precise function of RNase1 glycosylation (e.g., in respect to vessel protection or infectious diseases), the uptake of RNase1 by neighboring cells via endocytosis, and also include the opportunity to use RNase1 knockout mice in prospective studies. Consequently, these future perspectives in concert with the current knowledge about RNase1 might improve and expand the development of potential therapeutic options to preserve RNase1 function and vascular integrity in context of pathologic EC inflammation.

## Author Contributions

KB designed the review, summarized current literature and progress, wrote the manuscript, and created the figure. BS provided constructive advice and scientific input. Both authors contributed to revision and finalization of the manuscript.

## Conflict of Interest

The authors declare that the research was conducted in the absence of any commercial or financial relationships that could be construed as a potential conflict of interest.

## Footnotes

1 https://www.who.int/news-room/fact-sheets/detail/cardiovascular-diseases-(cvds)

2 https://www.proteinatlas.org/ENSG00000129538-RNASE1/

## Abbreviations

Asn: asparagine
EC: endothelial cell
eRNA: extracellular RNA
HDAC: histone deacetylase
HUVEC: human umbilical vein endothelial cells
I/R: ischemic/reperfusion
IL: interleukin
MI: myocardial infarction
poly I:C: polyinosinic polycytidylic acid
RA: rheumatoid arthritis
RNase1: Ribonuclease 1
TNF-α: tumor necrosis factor alpha.
